# Efficient national surveillance for health-care-associated infections

**DOI:** 10.1186/s12889-015-2172-9

**Published:** 2015-08-28

**Authors:** B. A. D. van Bunnik, M. Ciccolini, C. L. Gibbons, G. Edwards, R. Fitzgerald, P. R. McAdam, M. J. Ward, I. F. Laurenson, M. E. J. Woolhouse

**Affiliations:** Centre for Immunity, Infection and Evolution, University of Edinburgh, Edinburgh, UK; Department of Medical Microbiology, University Medical Center Groningen, University of Groningen, Groningen, The Netherlands; Microbiology Department, Scottish MRSA Reference Laboratory, Glasgow, UK; The Roslin Institute and Edinburgh Infectious Diseases, University of Edinburgh, Edinburgh, UK; Scottish Mycobacteria Reference Laboratory, Department of Laboratory Medicine, Royal Infirmary of Edinburgh, Edinburgh, UK

## Abstract

**Background:**

Detecting novel healthcare-associated infections (HCAI) as early as possible is an important public health priority. However, there is currently no evidence base to guide the design of efficient and reliable surveillance systems. Here we address this issue in the context of a novel pathogen spreading primarily between hospitals through the movement of patients.

**Methods:**

Using a mathematical modelling approach we compare the current surveillance system for a HCAI that spreads primarily between hospitals due to patient movements as it is implemented in Scotland with a gold standard to determine if the current system is maximally efficient or whether it would be beneficial to alter the number and choice of hospitals in which to concentrate surveillance effort.

**Results:**

We validated our model by demonstrating that it accurately predicts the risk of meticillin-resistant *Staphylococcus aureus* bacteraemia cases in Scotland.

Using the 29 (out of 182) sentinel hospitals that currently contribute most of the national surveillance effort results in an average detection time of 117 days. A reduction in detection time to 87 days is possible by optimal selection of 29 hospitals. Alternatively, the same detection time (117 days) can be achieved using just 22 optimally selected hospitals. Increasing the number of sentinel hospitals to 38 (teaching and general hospitals) reduces detection time by 43 days; however decreasing the number to seven sentinel hospitals (teaching hospitals) increases detection time substantially to 268 days.

**Conclusions:**

Our results show that the current surveillance system as it is used in Scotland is not optimal in detecting novel pathogens when compared to a gold standard. However, efficiency gains are possible by better choice of sentinel hospitals, or by increasing the number of hospitals involved in surveillance. Similar studies could be used elsewhere to inform the design and implementation of efficient national, hospital-based surveillance systems that achieve rapid detection of novel HCAIs for minimal effort.

**Electronic supplementary material:**

The online version of this article (doi:10.1186/s12889-015-2172-9) contains supplementary material, which is available to authorized users.

## Background

Healthcare-associated infections (HCAI) are a major concern for hospitals worldwide [1, 2]. HCAIs can spread rapidly throughout a network of hospitals [3, 4]. Rapid intervention for such outbreaks is of major importance, firstly to keep the number of infected patients to a minimum and secondly because there are considerable costs associated with large disease outbreaks [5, 6]. Examples of HCAIs causing concern are those caused by both meticillin-susceptible *Staphylococcus aureus* (MSSA) and methicillin-resistant *Staphylococcus aureus* (MRSA) as well as multi drug resistant gram negative bacteria, glycopeptide-resistant enterococcus and *Clostridium difficile*.

The need for efficient and reliable surveillance for HCAIs is recognised internationally [7, 8], but different countries have adopted different approaches to the implementation of a surveillance system. For example, in Scotland there is the Scottish Healthcare Associated Infection Programme supported by Health Protection Scotland (HPS) and the Scottish MRSA Reference Laboratory [9] which collectively gather data and monitor trends in the number of MRSA bacteraemias as well as antimicrobial resistance and virulence profiles. Elsewhere in the UK, amongst other systems, there are the voluntary reporting of all bacteraemias, the mandatory MRSA bacteraemia surveillance and the British Society for Antimicrobial Chemotherapy Resistance Surveillance Project [10]. Further examples are the European Antimicrobial Resistance Surveillance Network (EARS-Net) in Europe [11], the National Healthcare Safety Network (NHSN) in the United States of America [12] and the National Surveillance Initiative in Australia [13]. Unfortunately, there is not a good evidence base to guide the design of national surveillance programmes.

In this study, we assess the current laboratory based surveillance system for hospitals in Scotland in terms of efficiency in detecting a novel outbreak of an HCAI spread predominantly by direct patient contact and movement of patients between health care facilities, measured as the time to first detection and as the number of hospitals affected before detection. Efficiency is important because a more efficient surveillance system would detect outbreaks at an earlier stage when fewer hospitals and fewer patients are affected by an HCAI and decrease expenditure because fewer patients have to be treated.

Previous studies show that movement of patients is likely to play an important role in the spread of HCAIs as the patients act as vectors carrying pathogens from one hospital to the next [14–21].

Therefore, we use a recently developed mathematical model [22] to simulate the spread of a novel HCAI spread predominantly by direct patient movement between Scottish hospitals as a result of patient movements, i.e. with little or no spread in the community, and extend it by comparing existing surveillance programs with a (putative) optimal program to see if, with easily acquired information on the network of patient transfers, existing national surveillance schemes can be made more efficient. The model enables us to prioritise hospitals for inclusion in a laboratory surveillance system and thereby to address two key questions: 1) What is the optimal distribution of surveillance effort across hospitals and is the current system maximally efficient; and 2) Would there be benefits from increasing or decreasing the number of hospitals engaged in surveillance?

## Methods

### Patient movement data and the hospital network

For the purposes of this study, a “hospital” was defined as: a secondary healthcare facility with at least one inpatient per year and with at least one hospital specialty or department. Scottish hospitals were identified from those listed in Information Services Division (ISD) cost book reports for the financial year 2007-08 (R020 and R020LS, http://www.isdscotlandarchive.scot.nhs.uk/isd/6058.html) and were classified according to the terminology used by the ISD.

To quantify movements of patients between these hospitals we used patient admission data obtained on request from the ISD (http://www.isdscotland.org/). The patient admission data covered all admissions to healthcare facilities in Scotland between 01-01-2007 and 31-12-2007. From this dataset we extracted movements of patients between hospitals as either direct transfers, i.e. from one hospital directly to another hospital, or indirect transfers, i.e. when a patient is discharged from one hospital into the community and later (within the period covered by the data) admitted to another hospital.

We derived a movement matrix for all connected hospitals in Scotland by calculating for every hospital pair (i,j), the number of patients being moved from hospital j to hospital i, here denoted as w_i,j_, resulting in a N×N matrix of movements between hospitals, with N being the total number of hospitals that have connections with other hospitals.

From the patient admission dataset, movements between pairs of hospitals were identified for 182 unique hospitals (as defined above). Table 1 shows the types, as defined in the ISD costs book, and numbers of hospitals that were included in the analysis.

**Table 1:** Hospital types and number of occurrences of a particular type

Hospital Type	Count
Community hospitals	57
Large general hospitals	18
Long stay/psychiatric hospitals	16
Mental illness hospitals	14
Small long stay hospitals	14
General hospitals	13
Teaching hospitals	7
Long stay hospitals	6
Long stay/acute hospitals	6
Teaching mental illness hospitals	5
Learning disabilities hospitals	3
Sick children’s hospitals	3
Dental hospitals	2
Maternity hospitals	1
Other	17

### Bacteraemia case data

*Staphylococcus aureus* bacteraemia case data (number of MRSA and MSSA bacteraemia cases) for the year 2007 and stratified by hospital, were provided by Health Protection Scotland (HPS), NHS National Services Scotland, Glasgow, Scotland, UK. PAC 54/11. Caldicott approval was gained for this project and for the release of these data through HPS. From this dataset, the bacteraemia case data for the 182 hospitals that were included in the movement matrix were extracted and used in subsequent analysis.

### Current surveillance system in Scotland

The current surveillance system in Scotland consists of National Health Service (NHS) laboratories that receive specimens from the hospital or hospitals that they are associated with, mostly larger hospitals but some also cover up to five smaller, local hospitals. All laboratories report notifiable and other specified alert organisms to HPS. Those hospitals that do not have a laboratory send their specimens to another nearby hospital. Of the 182 hospitals in our study, 29 were associated with an NHS laboratory.

### Prioritising schemes

Three different prioritising schemes were used to assess both the current surveillance system in Scotland and two potentially better performing schemes. For the current surveillance system in Scotland we used the 29 hospitals with NHS laboratories according to their surveillance activity, i.e. hospitals that sent samples in 2007, we call this scheme “currently surveillance active”. Furthermore, we used a prioritising scheme based on the hospital category a hospital belonged to. Therefore we used either only teaching hospitals (seven in total in our network) or a combination of teaching hospital, large general hospitals and general hospitals (denoted as T + LG + G in figures and tables). Lastly, as a gold standard, we used a so-called greedy algorithm. Table 2 shows the median hospital size and the median number of patients received for the hospitals in the different prioritising schemes. Full details of how this greedy algorithm works can be found in [22] but, briefly, a greedy algorithm selects the first hospital to include as a sentinel hospital for surveillance based on the lowest average time to first infection (detection time) over all simulations. The next hospital is chosen in such a way that it minimises the average detection time when this hospital is added to the already existing list of sentinel hospitals. The greedy algorithm thus generates a putative optimal ordering of hospitals to be included in a national surveillance system.

**Table 2:** Median hospital size and number of patients received for the different priority lists. For hospital size the number of occupied bed days in the year 2007 was used as a proxy

Priority list	Hospitals included	Hospital size median (90 % range)	Patients received median (90 % range)
Surveillance active	29	124,824 (17,776 – 282,595)	2925 (389 – 7746)
Teaching hospitals	7	261,207 (188,894 – 322,494)	6759 (3806 – 9517)
T + LG + G	38	109,819 (9649 – 263,346)	2820 (294 – 6858)

### Mathematical model

Our modelling approach followed Ciccolini et al. [22] Briefly, we formulated a stochastic, discrete-time, Susceptible-Infectious model to simulate the spread of the pathogen between hospitals in the network. Three relevant assumptions of the model are that the pathogen spreads between hospitals predominately as a result of patient movements (as defined above), that it spreads much faster within a hospital than between hospitals, and that once infection is present in a hospital it remains there throughout the period of interest. A full discussion of these and other model assumptions is provided in the original publication [22].

To transmit the pathogen from one hospital to the next, a patient has to be a carrier of the pathogen when discharged, remain colonised or infected with the pathogen until being admitted to the next hospital, and then transfer the pathogen to another patient within this (subsequent) hospital. We combined the individual probabilities of these events into a single value, β, the probability of transmission per patient movement.

Combining the per patient probability of transmission and the number of referrals to a hospital, the probability of a hospital becoming infected and infectious can be written as:

$$ {P}_i^{S\to I}(t)=1-{\displaystyle \prod_{j:{H}_j\in I}}{\left(1-\beta \right)}^{w_{i,j}\Delta t} $$

Here *P*^*S* → *I*^(*t*) is the probability of a susceptible hospital becoming infected by an infectious hospital at time *t*, during one time-step *Δt* (*Δt* = 1 day was used throughout this paper), *I* is the set of all hospitals that are currently infectious, β is the per patient probability of transmission and *w*_*i,j*_ is the rate at which patient move from hospital *j* to hospital *i*. Thus, we calculate the contribution to the probability of infection of all currently (i.e. at time point *t*) infectious hospitals that transfer patients to hospital *i*. One of the assumptions of this model is that the parameters do not change over time or vary with hospital characteristics, such as hospital size. Other studies have shown that MRSA prevalence might be higher in larger hospitals [23, 24]. However we did not take this into account in our analyses.

### Simulations

To assess the performance of the different prioritisation schemes for surveillance the model was used to simulate the spread of a pathogen in a network of hospitals. A simulation time of 10 years was used throughout. The index hospital was uniform randomly picked from the list of available hospitals (see Additional file 1 for alternative choices of index hospital). Data on the status of each hospital on each day of the simulation and the time to first infection obtained for each hospital were recorded. All simulations for the comparison of the different surveillance strategies were performed using baseline values β = 0.001. This value was used by Ciccolini et al. [22] and reproduces the observed increase in hospital-level prevalence of EMRSA-15 and EMRSA-16 in England and Wales between 1992 and 1997 [25]. For surveillance purposes we assume that an (bloodstream) infection is identified immediately and that there is no difference between hospitals in times from infection to detection. Response to infection is not explicitly included in the model although control measures applied across all infected hospitals and prevention measures applied across all uninfected hospitals are implicit in the value of the parameter β.

### Model fit

As a model-predicted risk estimate we used the reciprocal of the average time to first infection $\left(\overline{t_{inf}}\right)$ averaged over the 20,000 simulations. To test how well HCAI risk, as predicted by the model, agreed with empirical data on MRSA bacteraemia a Receiver Operating Characteristic (ROC) analysis (see Additional file 1 for details on the analysis) was employed. We calculated the area under the ROC curve (AUC), which provides a measure of a model’s ability to discriminate between hospitals that had MRSA bacteraemia cases, and those that did not [26]. We note that bacteraemia data do not capture asymptomatic carriage, but we assume that these are correlated.

### Impact of non-participating hospitals

In order to test the importance of full participation of priority hospitals we studied the impact of removing hospitals from the surveillance program. We systematically removed from one up to six hospitals and determined the effects on the average time until first detection. We then took the worst case scenario, i.e. that subset of remaining hospitals that showed the maximum time until first detection.

### Costs estimation

For a comparison of the costs involved with disease surveillance we explored two options to estimate direct costs associated with the different sentinel surveillance schemes. For the first option it was assumed that there is a fixed cost per hospital for surveillance. The second option assumes that the costs are dependent on the number of patients a hospital receives from other hospitals in the network, either via direct transfers or via indirect transfers. It is assumed that all (or a set fraction of) incoming patients are screened and that the expense per patient is the same for all hospitals and all patients. The fixed and variable approaches were calibrated to give the same total cost when all hospitals were included.

## Results

### Patient movement data

**Fig. 1:**
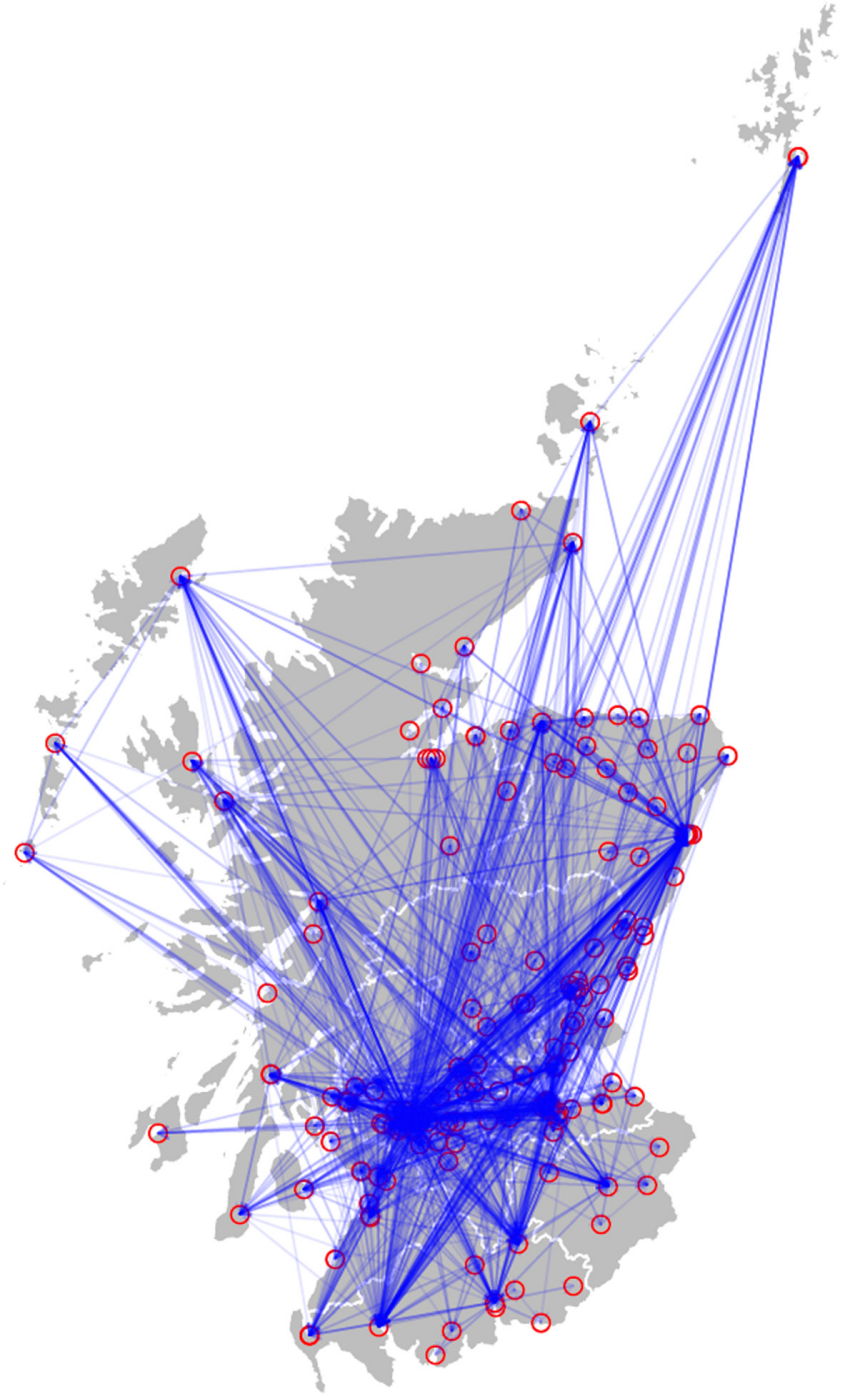
Hospitals included and their connections. Red open circles indicate a hospital and blue arrows indicate a connection (at least one patient transferred) between the two hospitals

From the patient admissions data for 2007, 182 hospitals admitted patients who had previously been discharged from a Scottish hospital within that year or discharged patients who were subsequently admitted to another hospital within Scotland in that year. Theoretically, this could have led to 32,942 connections if all hospitals were connected to all other hospitals. However, we identified just 2360 connections (7.1 % of those possible). Figure 1 shows a map of the hospitals included and the connections between them (a line between two hospitals represents movement between the two connected hospitals, in either direction). Furthermore we found that patients who had spent time in the community between stays at two different hospitals did so on average for a median 19 days; Additional file 1: Figure S1 shows the distribution of time spent in the community between subsequent admissions.

### Simulation results

There was considerable variation (over the 20,000 simulations) in the average time to infection for individual hospitals predicted by the model, with modal times ranging from 40 days to 3520 (Additional file 1: Figure S2).

**Fig. 2:**
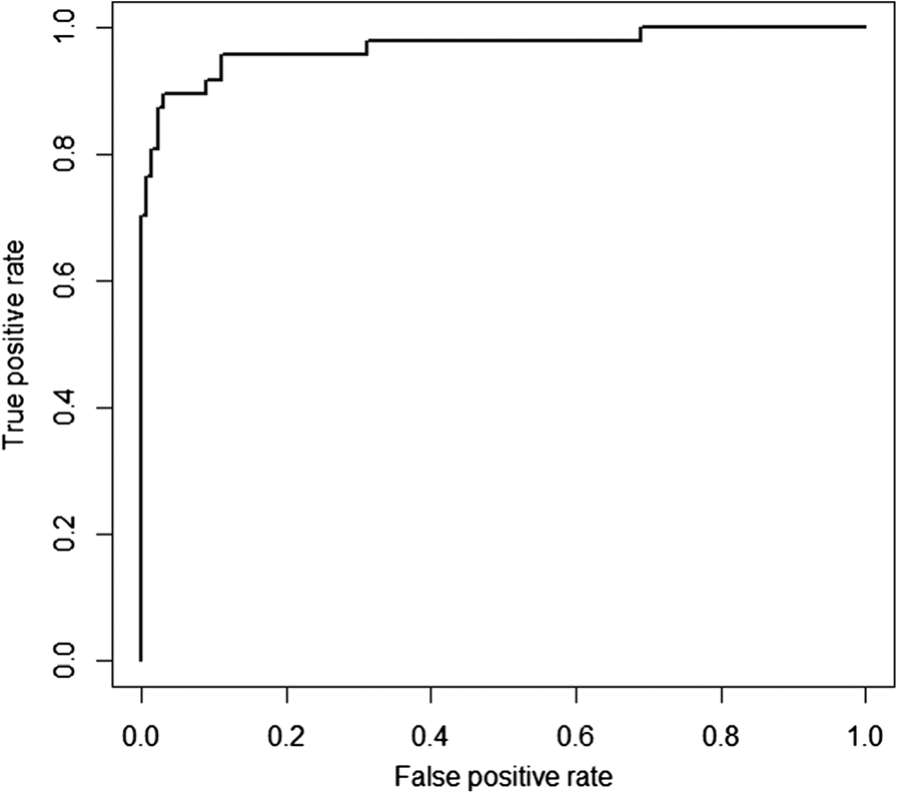
The ROC curve for the comparison between bacteraemia cases in Scotland and predictions of the stochastic network model. The x-axis shows the false positive rate and the y-axis shows the true positive rate. The AUC = 0·97

The results of the ROC analyses are shown in Fig. 2. The AUC is 0.97, indicating a very good fit of the model prediction to data on MRSA bacteraemia cases in Scotland. If we compare this to another measure as a predictor for MRSA bacteraemia cases, hospital size (measured as the number of staffed beds in a hospital), it is clear that our model fits the bacteraemia case data better than hospital size, which has an AUC of 0.88 (Additional file 1: Figure S3).

**Fig. 3:**
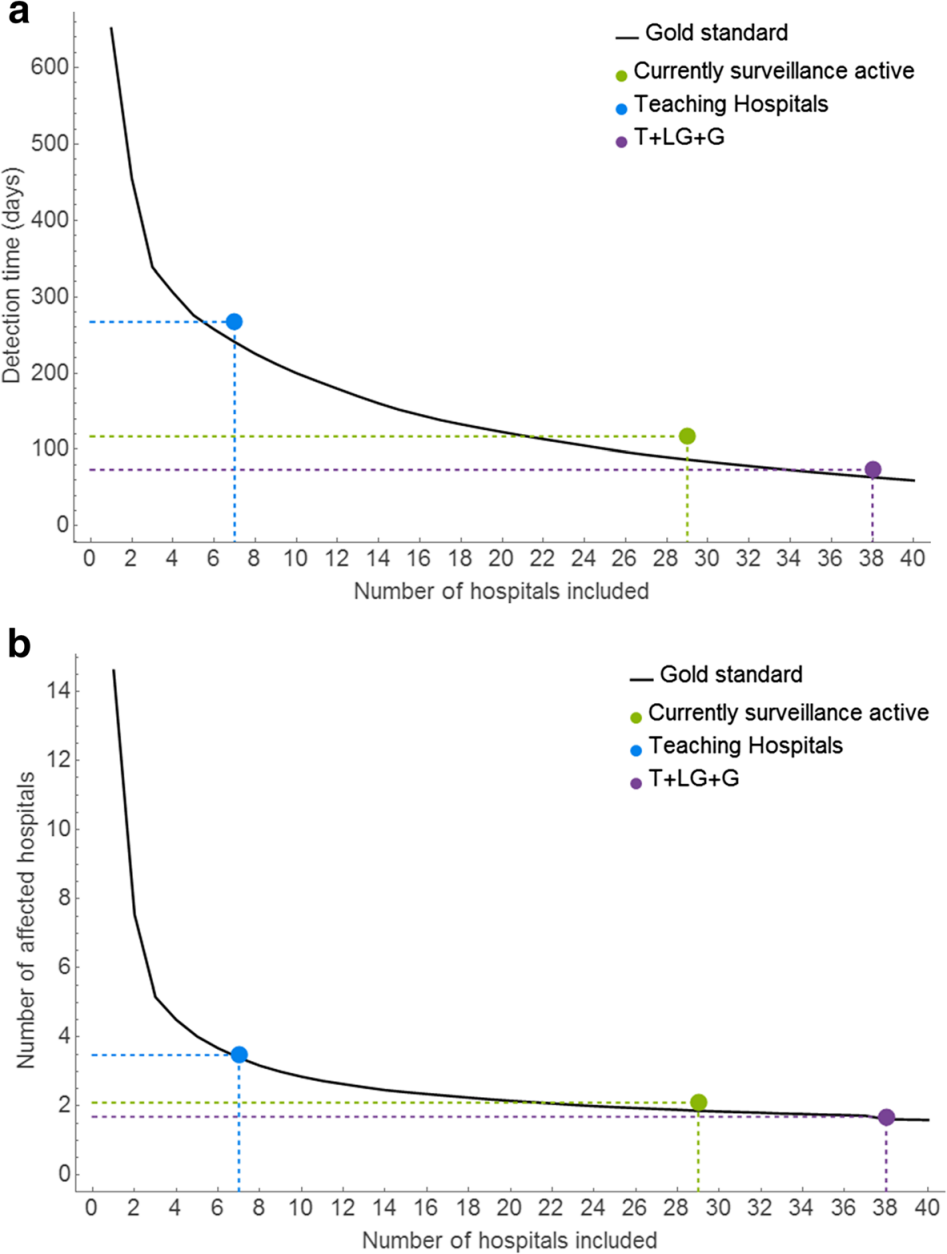
**a**. Sentinel surveillance system performance. Average detection time of a novel HCAI, following emergence in a single randomly selected hospital versus number of hospitals participating in surveillance. The solid black line corresponds to the gold standard ‘greedy’ algorithm; the coloured points indicate the average detection time after including all hospitals in that particular list. Dashed coloured lines are plotted as reference lines. **b**. As 3a but showing the average number of affected hospitals

We compared the performance of the different methods to create the priority list for a sentinel surveillance system by two different measures: the detection time and the number of hospitals affected before detection. Figure 3a shows the average detection time for the different prioritisation methods against the fraction of hospitals included in the surveillance system. Similarly, Fig. 3b shows the average number of affected hospitals before detection in the fraction of sentinel hospitals. The results for the gold standard and the three other options (currently surveillance active, teaching hospitals only or teaching hospitals + large general hospitals + general hospitals) are also indicated in Fig. 3b. Detection times and number of affected hospitals at detection was calculated, for the above three options, by employing all the hospitals participating in the surveillance programme. Table 3 shows the results in terms of number of hospitals included and detection times of each method compared to the gold standard.

**Table 3:** Detection for the different priority lists when all hospitals of that particular list are included as sentinel hospitals. Gold standard hospitals needed indicates the number of hospitals that would be needed using the gold standard algorithm to detect an outbreak in the same time as using the priority list. Gold standard detection time indicates the detection time needed with the greedy algorithm if the same number of hospitals would be included from the priority list based on the Gold standard algorithm

Priority list	Hospitals included	Detection time (days)	Gold standard hospitals needed	Gold standard detection time (days)
Surveillance active	29	117	22	87
Teaching hospitals	7	268	6	241
T + LG + G	38	74	34	64

This shows that if we include the currently surveillance active hospitals (*N* = 29) this results in an average detection time of 117 days (90^th^ percentile: 308 days), whilst optimal selection of 29 hospitals (i.e. from the gold standard) would result in detecting the outbreak 30 days earlier (87 days, 90^th^ percentile: 260 days) (see also Fig. 3a). Alternatively, optimal selection to detect the outbreak within 117 days would only need 22 hospitals. If we include only the seven teaching hospitals as sentinel hospitals in a surveillance scheme average detection time would be 268 days (90^th^ percentile: 595 days), an increase of 151 days.

The average number of hospitals affected before detection is 2.1 (90^th^ percentile: 3 hospitals) for the currently surveillance active hospitals, compared to 3.4 (90^th^ percentile: 5 hospitals) if only teaching hospitals are included. If the number of hospitals to include as sentinels for surveillance is extended to all 38 teaching, large general and general hospitals then the average detection time is 74 days (90^th^ percentile: 280 days), a decrease of 43 days compared to the currently surveillance active hospitals. Similarly, the average number of hospitals affected decreased by 0.4 hospital to 1.7 (90^th^ percentile: 2) affected hospitals.

**Fig. 4:**
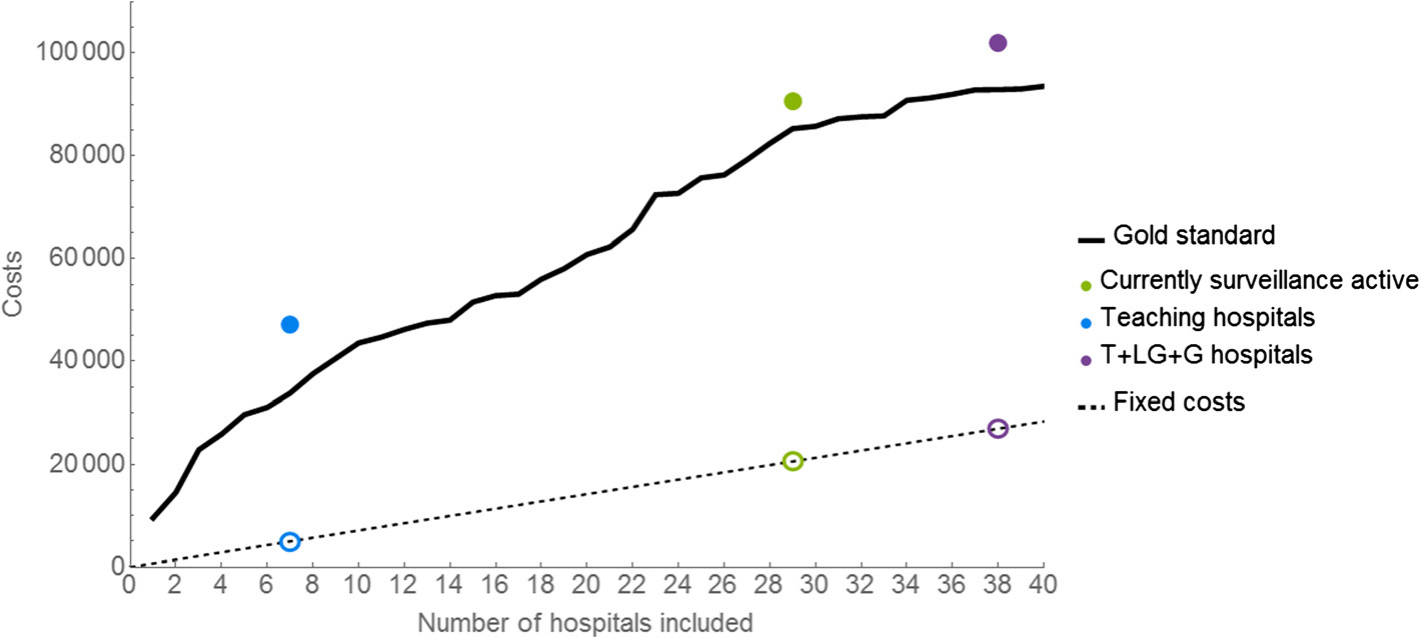
Costs associated with the different prioritising methods. Total cost associated with the gold standard selection of hospitals (lines) is compared to three other selection criteria (symbols). Costs are calculated either as a fixed cost per hospital (dashed line, open symbols) or variable costs that depend on the number of patient in-movements (see main text) (solid line, closed symbols). Costs are in arbitrary units but fixed and variable costs are scaled to give the same total cost if all hospitals participate in surveillance

The relative costs associated with different surveillance schemes are depicted in Fig. 4. Two limiting cases are illustrated: i) there is a fixed cost per hospital, so total cost is proportional to the number of hospitals included; and ii) costs are proportional to the number of patient in-movements (from other hospitals, directly or indirectly) per hospital. The actual costs will be a combination of fixed and pro rata costs and so are likely to fall between these two extremes. Fixed costs, by definition, rise linearly with the number of hospitals participating, but are independent of the choice of hospitals. Variable costs, however, tend to rise faster initially because larger hospitals with more in-movements have higher priority, and are dependent on the exact choice of hospitals. For variable costs, the currently surveillance active hospitals (green symbols in Fig. 4) not only have greater than optimal detection time (Fig. 3a) but are also more costly. Optimal selection of hospitals would therefore both improve the performance of the surveillance system and reduce costs.

## Discussion

In this study, we have constructed a network of all hospitals in Scotland that are connected through the movements of patients. Outbreaks of an HCAI were simulated over this network in order to identify those hospitals that are most prone to being infected with an emerging pathogen spread by patient movements and to assess different prioritisations of hospitals for inclusion in surveillance systems for early detection of a novel pathogen spreading between hospitals. In Scotland, the vast majority of MRSA bacteraemia cases are caused by the strains EMRSA-15 and EMRSA-16, both of which are strongly associated with hospitals [27]. Therefore we tested how well the risk, as predicted by the model, fitted with bacteraemia case-data using an ROC analysis which shows that the model accurately predicts the risk of a hospital being affected (Fig. 2).

We assessed the performance of the current laboratory based surveillance system as it is implemented in Scotland as well as two other possible surveillance schemes and a putative optimal surveillance scheme. Our results show that if hospitals are prioritised on their surveillance activity, as indicated by whether they submitted samples to the SMRSARL in 2007 (*N* = 29), the average detection time before detection is significantly above the optimum, although the expected number of hospitals affected is near the optimum. However, if fewer hospitals were included in a surveillance scheme, i.e. only teaching hospitals (*N* = 7), this would increase the average time to first detection by about 150 days (Fig. 3a and Table 3). If, on the other hand, more hospitals were included, i.e. all teaching, large general and general hospitals (*N* = 38), this would decrease the average detection time by 43 days. However, our results do reveal diminishing returns: as more hospitals are included, less is gained in terms of detection time or number of affected hospitals (Fig. 3a and b). The number of hospitals to be included will ultimately be dictated by what is judged an ‘acceptable’ time before detection, although that number is minimised by using the methodology described here. That decision will depend on a number of factors, such as the cost of treating an infection, the risk of onward spread and the need for hospital-wide preventive measures.

Furthermore, Fig. 4 shows that optimal selection of hospitals to be included in a sentinel surveillance system can also reduce direct cost of surveillance. Here we have used a very simple estimate of the costs associated with surveillance; we assume that all or a fixed proportion of patients who have been at another hospital within the past 12 months are screened clinically and, if indicated, by culture upon arrival and that these costs linearly increase with each patient. In practice, hospitals in Scotland currently use “clinical risk assessment” (CRA) to assess patients that enter the hospital. Asymptomatic patients are not screened automatically for bacteraemia by taking blood cultures, but surveillance swabs for the detection of MRSA carriage may be taken depending on the CRA assessment outcome [9].

This study has some limitations that should be considered. We assumed that all affected hospitals have detectable infections (using MRSA bacteraemia as an exemplar). Affected hospitals with only asymptomatically colonized patients would either be missed or detection delayed until bacteraemia cases appear. However, the original model [22] was calibrated using case data for bloodstream MRSA infections and not on colonisation case data, so this is already taken into account. Furthermore we chose a specific value for β consistent with the observed rate of spread of MRSA in England [22]. A sensitivity analysis shows that our results and conclusions are robust to changes in the value for β. Although the speed with which the disease spreads over the network is influenced by β, the results (in terms of efficiency of existing surveillance schemes compared to a gold standard) do not change significantly (see Additional file 1: Figure S6 for sensitivity analysis results). Moreover these simulations show that for higher values of β the need for an efficient surveillance system is even more important as there is less time to detect an (emerging) infection.

Lastly, the results found in this study depend on the correctness of the model and all implicit and explicit assumptions that were made. We showed that the risk as predicted by the model is fitting well to empirical data, which strengthens confidence in the model and its assumptions.

As mentioned before, hospitals that do not have a microbiology laboratory may send their samples to a nearby (large) hospital, and this makes those larger hospitals even more important for surveillance purposes (and also making the current system more robust). If only a few large hospitals are included in the sentinel surveillance scheme (i.e. teaching hospitals only) then not only do detection times increase but the non-participation of just one of the hospitals could have a significant impact on the time to first detection (Additional file 1: Figure S4). Surveillance systems that include more hospitals tend to be more robust to non-participation.

## Conclusion

To conclude, in this paper we have demonstrated that the current laboratory based surveillance system as it is used in Scotland to detect novel pathogens spreading by means of patient movements is less efficient than a putative optimal surveillance system in time to first detection of a HCAI. We have shown that this time until first detection can be reduced either by increasing the number of hospitals participating in surveillance or by optimally selecting which hospitals to include in a surveillance system. Similar studies elsewhere could be used to inform national health care services on the design and implementation of efficient surveillance systems that achieve rapid detection of novel HCAIs for minimal effort.

## Additional file

Additional file 1:
**Additional information.** (PDF 384 kb)

## References

[1] Stein RA (2011). Antibiotic Resistance: A Global, Interdisciplinary Concern. Am Biol Teach.

[2] Barbut F, Jones G, Eckert C (2011). Epidemiology and control of Clostridium difficile infections in healthcare settings: an update. Curr Opin Infect Dis.

[3] Won SY, Munoz-Price LS, Lolans K, Hota B, Weinstein RA, Hayden MK (2011). Emergence and Rapid Regional Spread of Klebsiella pneumoniae Carbapenemase-Producing Enterobacteriaceae. Clin Infect Dis.

[4] Pearman JW (2006). 2004 Lowbury Lecture: the Western Australian experience with vancomycin-resistant enterococci - from disaster to ongoing control. J Hosp Infect.

[5] Bouza E (2012). Consequences of Clostridium difficile infection: understanding the healthcare burden. Clin Microbiol Infect.

[6] Goetghebeur M, Landry PA, Han D, Vicente C (2007). Methicillin-resistant Staphylococcus aureus: A public health issue with economic consequences. Can J Infect Dis Med Microbiol.

[7] Ducel GF J, Nicolle L (2002). Prevention of hospital-acquired infections: a practical guide. World Health Organization Dept of Epidemic and Pandemic Alert and Response.

[8] WHO (2011). Report on the burden of endemic health care-associated infection worldwide.

[9] Protocol for CRA MRSA Screening National Rollout in Scotland [http://www.documents.hps.scot.nhs.uk/hai/mrsa-screening/mrsa-screening-staff-implementation-protocol-v1.7-2013-01-31.pdf]. Accessed 24 August 2015.

[10] Reynolds R (2009). Antimicrobial resistance in the UK and Ireland. J Antimicrob Chemother.

[11] European Antimicrobial Resistance Surveillance Network [http://www.ecdc.europa.eu/en/activities/surveillance/EARS-Net/Pages/index.aspx]. Accessed 24 August 2015.

[12] Dykhuizen D (1998). Santa Rosalia revisited: Why are there so many species of bacteria?. Antonie Van Leeuwenhoek.

[13] National Safety and Quality Health Service [http://www.safetyandquality.gov.au/our-work/healthcare-associated-infection/national-hai-surveillance-initiative/]. Accessed 24 August 2015.

[14] Robotham JV, Scarff CA, Jenkins DR, Medley GF (2007). Meticillin-resistant Staphylococcus aureus (MRSA) in hospitals and the community: model predictions based on the UK situation. J Hosp Infect.

[15] Widerstrom M, Monsen T, Karlsson C, Wistrom J (2006). Molecular epidemiology of meticillin-resistant coagulase-negative staphylococci in a Swedish county hospital: evidence of intra- and interhospital clonal spread. J Hosp Infect.

[16] Cordeiro JCR, Silbert S, Reis AO, Sader HS (2004). Inter-hospital dissemination of glycopeptide-resistant Enterococcus faecalis in Brazil. Clin Microbiol Infect.

[17] Schaefler S, Jones D, Perry W, Baradet T, Mayr E, Rampersad C (1984). Methicillin-resistant Staphylococcus aureus strains in New York City hospitals: inter-hospital spread of resistant strains of type 88. J Clin Microbiol.

[18] Eveillard M, Quenon JL, Rufat P, Mangeol A, Fauvelle F (2001). Association between hospital-acquired infections and patients’ transfers. Infect Control Hosp Epidemiol.

[19] Donker T, Wallinga J, Grundmann H (2010). Patient referral patterns and the spread of hospital-acquired infections through National Health Care Networks. Plos Comput Biol.

[20] Lesosky M, McGeer A, Simor A, Green K, Low DE, Raboud J (2011). Effect of patterns of transferring patients among healthcare institutions on rates of nosocomial methicillin-resistant staphylococcus aureus transmission: a Monte Carlo simulation. Infect Control Hosp Epidemiol.

[21] Austin DJ, Anderson RM (1999). Transmission dynamics of epidemic methicillin-resistant Staphylococcus aureus and vancomycin resistant enterococci in England and Wales. J Infect Dis.

[22] Ciccolini M, Donker T, Grundmann H, Bonten MJM, Woolhouse MEJ (2014). Efficient surveillance for healthcare-associated infections spreading between hospitals. Proc Natl Acad Sci.

[23] Livermore DM, Pearson A (2007). Antibiotic resistance: location, location, location. Clin Microbiol Infect.

[24] Tiemersma EW, Bronzwaer S, Lyytikainen O, Degener JE, Schrijnemakers P, Bruinsma N, Monen J, Witte W, Grundmann H, European Anti Resis Sur Sys P (2004). Methicillin-resistant Staphylococcus aureus in Europe, 1999–2002. Emerg Infect Dis.

[25] Epidemic Methicilin-resistant Staphylococcus aureus [http://webarchive.nationalarchives.gov.uk/20140714084352/http://www.hpa.org.uk/CDR/archives/1997/cdr2297.pdf. Accessed 24 August 2015.

[26] Dohoo IR, Martin SW, Stryhn H. Veterinary Epidemiologic Research. 2nd edn. VER Inc., Charlottetown, Prince Edward Island: Canada; 2009

[27] McAdam PR, Templeton KE, Edwards GF, Holden MTG, Feil EJ, Aanensen DM, Bargawi HJA, Spratt BG, Bentley SD, Parkhill J (2012). Molecular tracing of the emergence, adaptation, and transmission of hospital-associated methicillin-resistant Staphylococcus aureus. Proc Natl Acad Sci U S A.

