# Supplementary material for: Efficient national surveillance for health-care-associated infections
Source: BMC Public Health. 2015 Aug 28;15:832. doi: 10.1186/s12889-015-2172-9 (PMC4552460; doi:10.1186/s12889-015-2172-9)
Supplement: Additional file 1: — Additional information. (PDF 384 kb) [file 12889_2015_2172_MOESM1_ESM.pdf]

Additional information

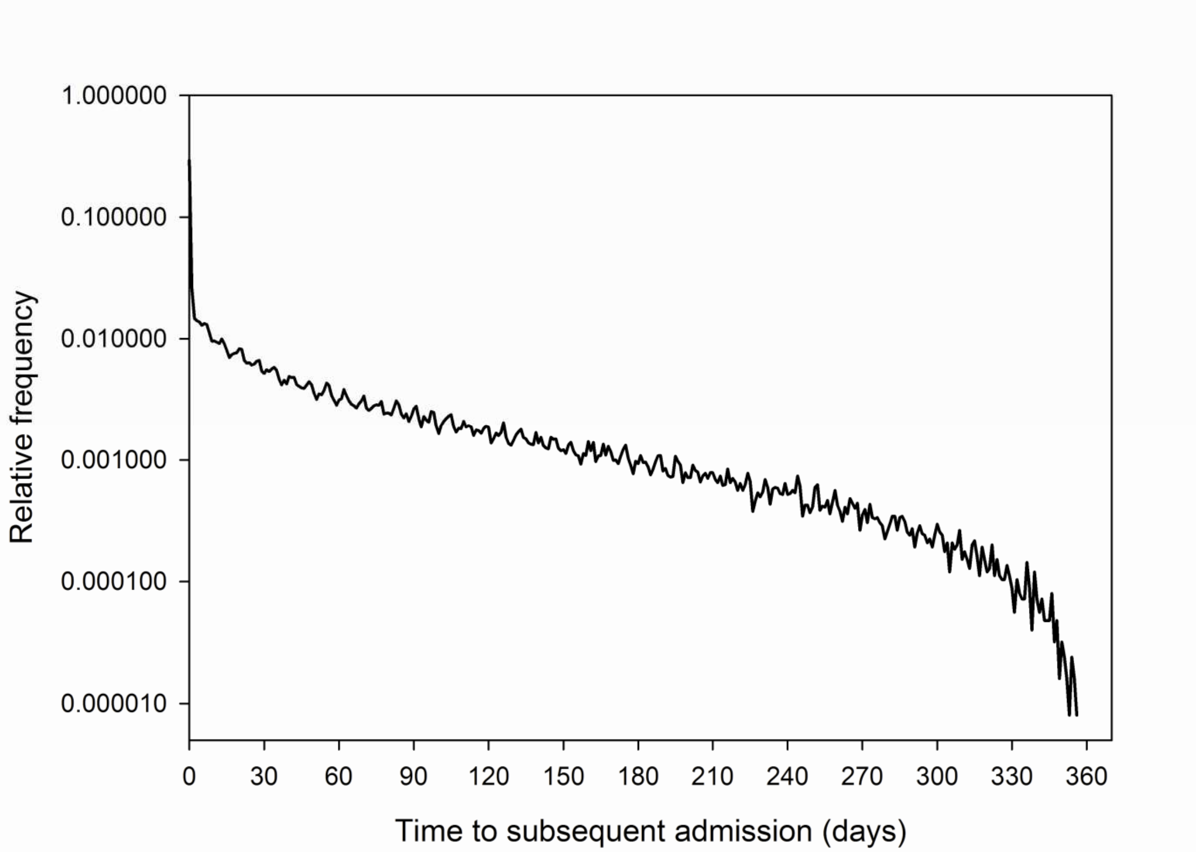

Figure S1. Relative frequency of the number of days spent outside the hospital between subsequent admissions.

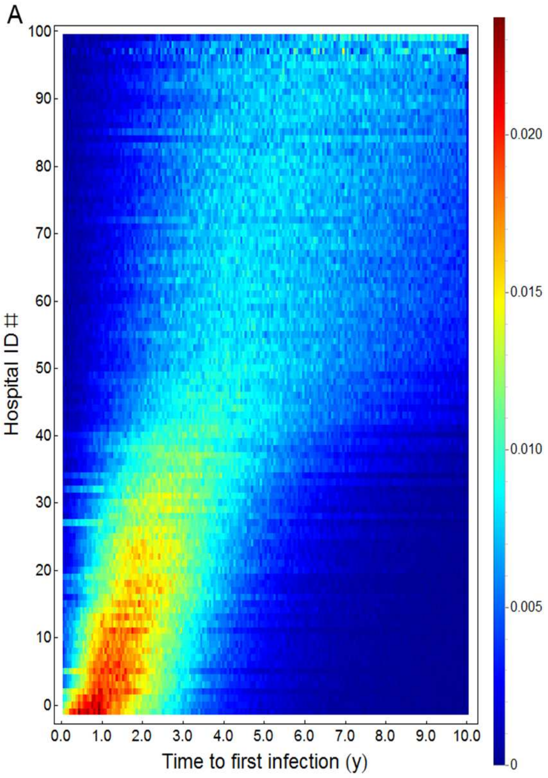

Figure S2. Time to first infection. Probability, estimated as the relative frequency of time to first infection, of individual hospitals in Scotland becoming affected by a novel HCAI, following introduction in a randomly selected hospital at time t=0. Results obtained in the baseline scenario with β=0.001 and for 20000 simulations. Hospitals have been sorted along the y-axis according to increasing value of median time to infection; the first 100 hospitals with the lowest median time to infection are plotted

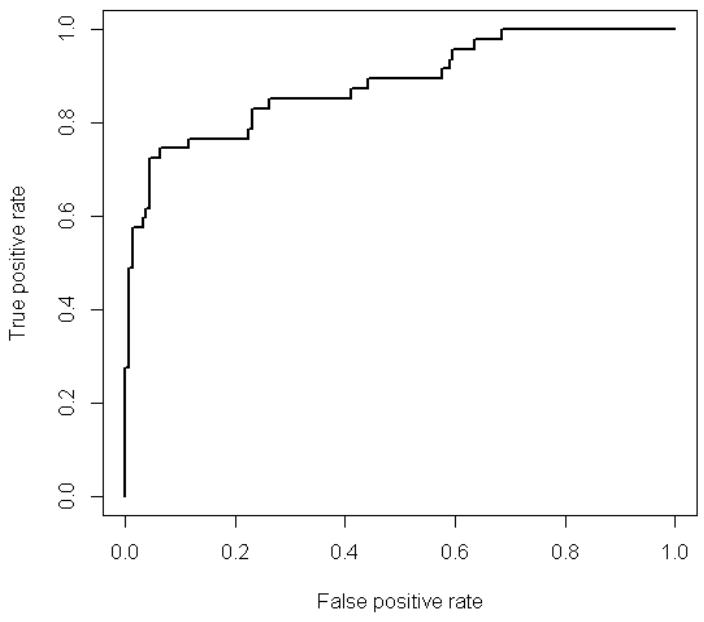

Figure S3. The ROC curve for the comparison between bacteraemia cases in Scotland and hospital size (measured by averaged staffed beds in 2007). The x-axis shows the false positive rate and the y-axis shows the true positive rate. The AUC is 0.88.

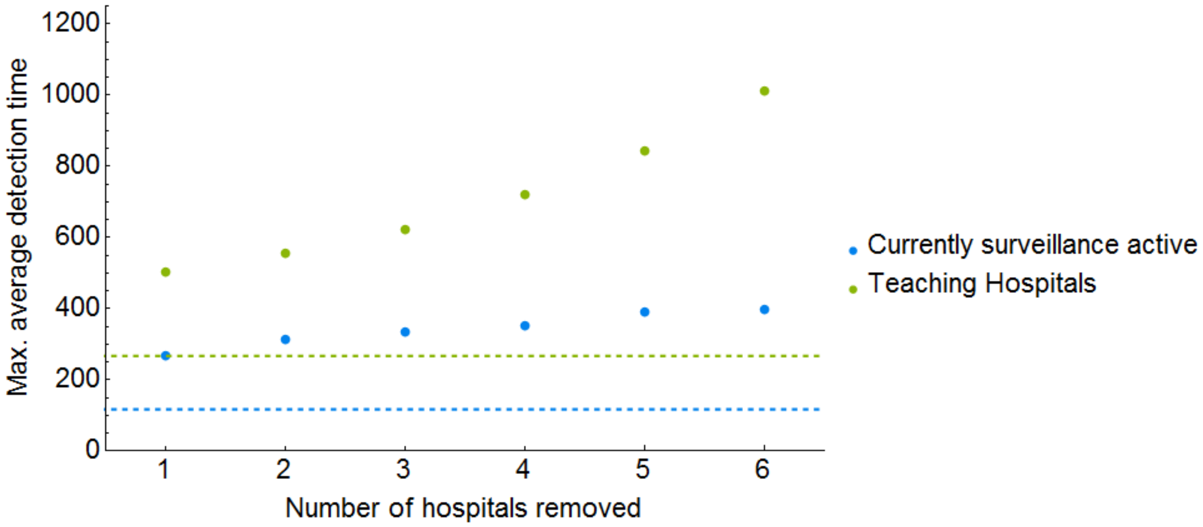

Figure S4. Detection times after removal of one or more hospitals from a particular priority list. The symbols represent the worst case scenario, i.e. that subset of remaining hospitals that showed the maximum time until first detection. The dashed lines indicate the average detection time when all hospitals from that list are included.

ROC analysis

For the (non-parametric) ROC analysis, the hospitals are arranged in the descending order of the predicted probability $(1 - \exp(-1 * day / \overline{t_{inf}})$, which acts as the cut-off point for distinguishing between positive and negative hospitals. In the bacteraemia dataset (see Methods section for more details), of the 182 hospitals included, 47 had bacteraemia cases (i.e. positive). For a given value of cut-off point, the false positive rate (FPR) is calculated as NFP/135 where NFP (=Number of False Positives) is the number of hospitals that are actually negative and have higher predicted probability than the cut-off point, the true positive rate (TPR) as NTP/47 where NTP (=Number of True Positives) is the number of hospitals that are actually positive and have a higher predicted probability than the cut-off point. The ROC curve is then obtained by plotting TPR versus FPR over all possible cut-off point values, from which the AUC can be derived.

Alternative choice of index hospital

For the simulations in the main paper we selected the index hospital at random. However alternative ways of index hospital selection do exist and here we show the results of randomly picking a hospital using a weighted distribution where the weight of a hospital was based on the size of the hospital, measured by the number of staffed beds per hospital in 2007. The rationale for this index selection is that larger hospitals have higher probability of generating their own index case and they are likely to receive more patients from outside the country and therefore have a higher probability of being an index case. Figure S5 shows the detection time (Figure S5A) and the number of affected hospitals before detection (Figure S5B) for the different surveillance scheme implementations. Table S1 shows the results of a comparison in terms of number of hospitals included and detection times of each method compared to the gold standard. For further explanation of the figures or table we refer to the main text.

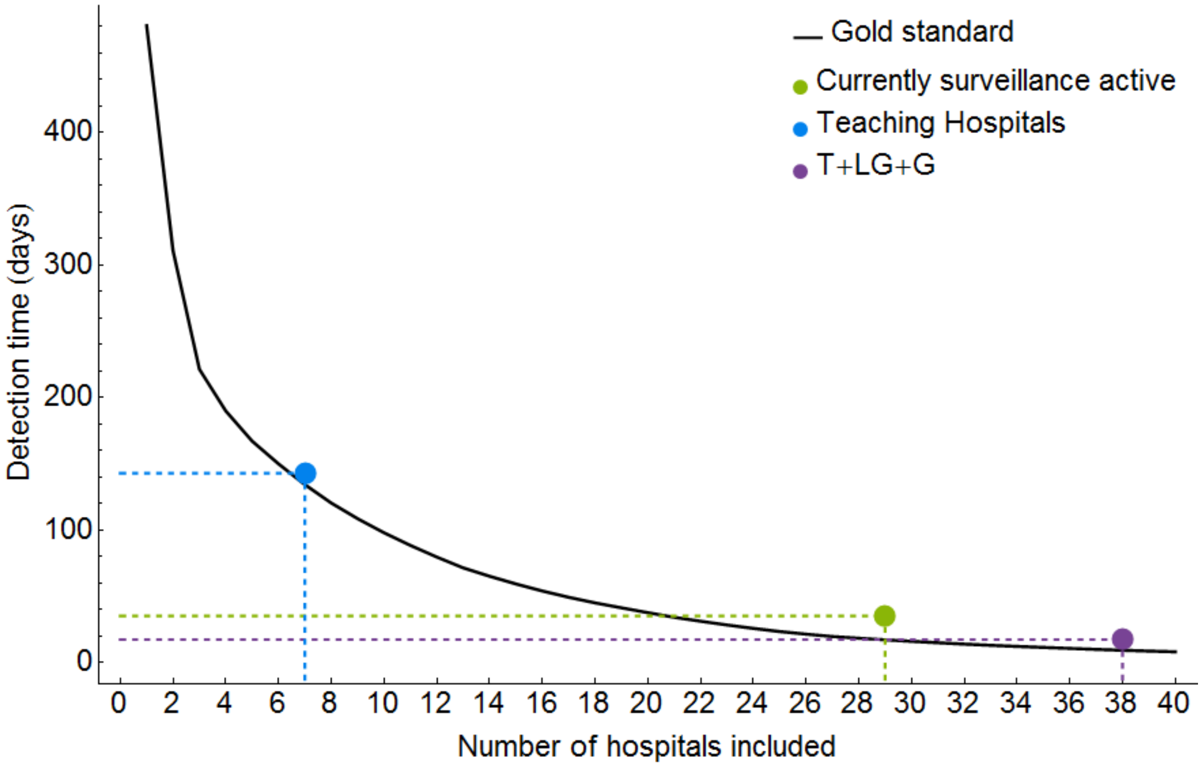

Figure S5A. Sentinel surveillance system. Average detection time of a novel HCAI, following emergence in a single weighted randomly selected hospital versus number of hospitals participating in a sentinel surveillance programme. The solid black line corresponds to the gold standard algorithm; the coloured symbols indicate the average detection time after including all hospitals in that particular list. Dashed coloured lines are plotted as reference lines.

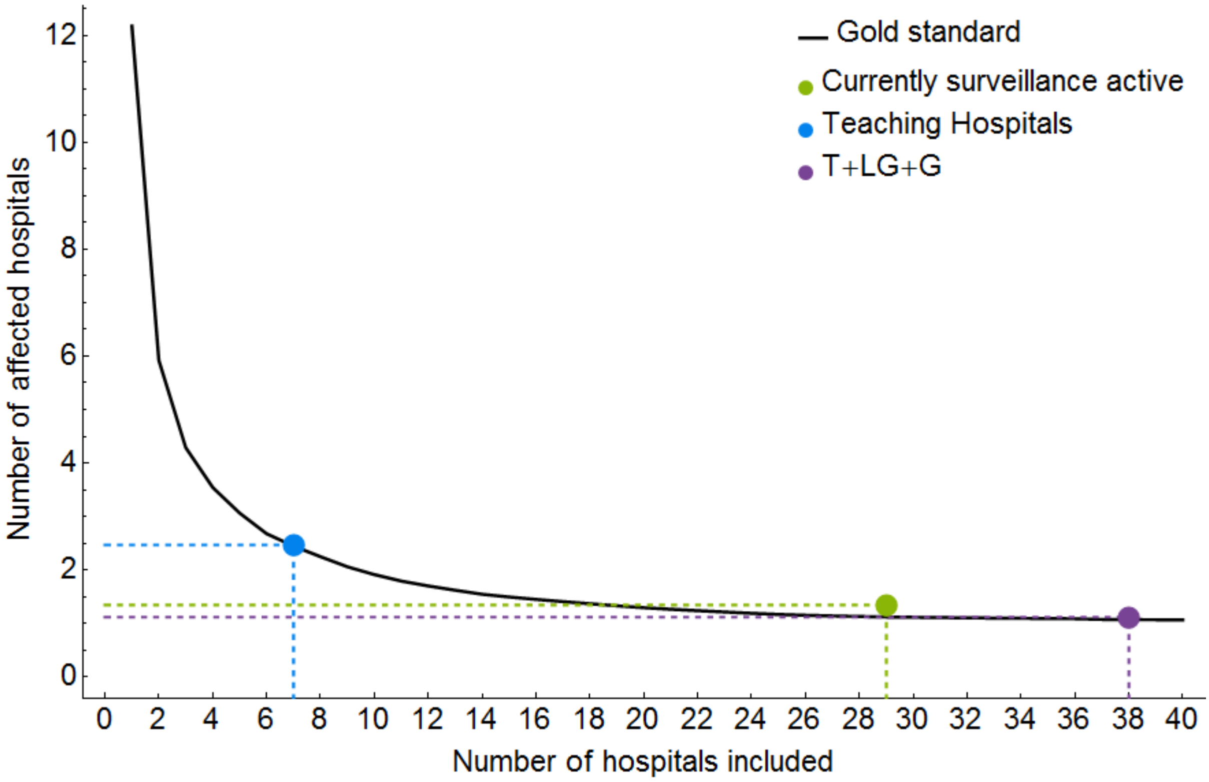

Figure S5B. Sentinel surveillance system performance. As S5A for the average number of affected hospitals before detection of a novel HCAI.

Table S1. Detection for the different priority lists when all hospitals in that particular list are included as sentinel hospitals. Gold standard hospitals needed indicates the number of hospitals that would be needed in the Gold standard algorithm to detect an outbreak in the same time as the priority list. Gold standard detection time indicates the detection time needed with the greedy algorithm if the same number of hospitals would be included from the priority list based on the gold standard algorithm.

Priority list	Hospitals included	Detection time (days)	Gold standard hospitals needed	Gold standard detection time (days)
**Surveillance active**	29	36	21	18
**Teaching hospitals**	7	143	7	135
**T+LG+G**	38	18	29	10

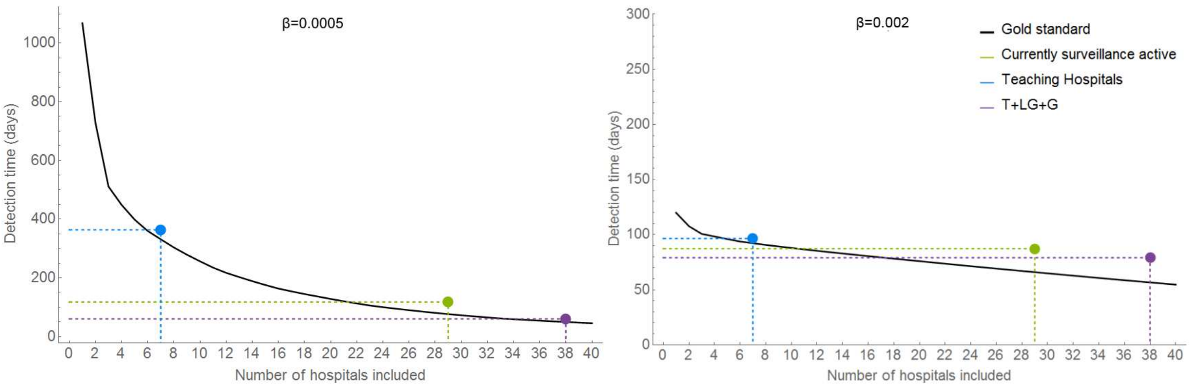

Figure S6. Sentinel surveillance system. Average detection time of a novel HCAI, following emergence in a single weighted randomly selected hospital versus number of hospitals participating in a sentinel surveillance programme for rescaled values of β (left panel, β=0.0005, right panel β=0.002) . The solid black line corresponds to the gold standard algorithm; the coloured symbols indicate the average detection time after including all hospitals in that particular list. Dashed coloured lines are plotted as reference lines.
